# Genome-wide association analysis for emergence of deeply sown rice (*Oryza sativa*) reveals novel aus-specific phytohormone candidate genes for adaptation to dry-direct seeding in the field

**DOI:** 10.3389/fpls.2023.1172816

**Published:** 2023-06-12

**Authors:** Sandeep A. Sakhale, Shailesh Yadav, Lindsay V. Clark, Alexander E. Lipka, Arvind Kumar, Erik J. Sacks

**Affiliations:** ^1^ Department of Crop Sciences, University of Illinois Urbana-Champaign, Urbana, IL, United States; ^2^ International Rice Research Institute (IRRI), Los Baños, Philippines; ^3^ International Rice Research Institute (IRRI), South Asia Regional Centre (ISARC), Varanasi, India; ^4^ Africa Rice Center (AfricaRice), Abidjan, Côte d’Ivoire; ^5^ Seattle Children’s Research Institute, Seattle, WA, United States

**Keywords:** dry direct-seeded rice (Dry-DSR), genome wide association study (GWAS), mixed linear model (MLM), quantitative trait loci (QTL), rice diversity panel 1 (RDP1), single nucleotide polymorphism (SNP), 3,000 rice genome project (3k RGP)

## Abstract

Dry direct-seeded rice (dry-DSR) is typically sown deeply to circumvent the need for irrigation, and thus seedling emergence is a crucial trait affecting plant stand and yield. To breed elite cultivars that use less water and are climate-resilient, an understanding of the genomic regions and underlying genes that confer emergence in deeply sown dry-DSR would be highly advantageous. A combined diversity panel of 470 rice accessions (RDP1 plus aus subset of 3K RGP) was evaluated with 2.9 million single nucleotide polymorphisms (SNPs) to identify associations with dry-DSR traits in the field and component traits in a controlled-environment experiment. Using genome-wide association study (GWAS) analyses, we identified 18 unique QTLs on chromosomes 1, 2, 4, 5, 6, 7, 9, 10, and 11, explaining phenotypic variance ranging from 2.6% to 17.8%. Three QTLs, namely, *qSOE-1.1*, *qEMERG-AUS-1.2*, and *qEMERG-AUS-7.1*, were co-located with previously reported QTLs for mesocotyl length. Among the identified QTLs, half were associated with the emergence of aus, and six were unique to the aus genetic group. Based on functional annotation, we identified eleven compelling candidate genes that primarily regulate phytohormone pathways such as cytokinin, auxin, gibberellic acid, and jasmonic acid. Prior studies indicated that these phytohormones play a critical role in mesocotyl length under deep sowing. This study provides new insight into the importance of aus and *indica* as desirable genetic resources to mine favorable alleles for deep-sowing tolerance in rice. The candidate genes and marker-tagged desirable alleles identified in this study should benefit rice breeding programs directly.

## 1 Introduction

Rice (*Oryza sativa*) is life for three billion people, supplying 35% to 60% of daily calories for nearly half of humanity (Fageria, 2003; FAO, 2017). Global rice demand is estimated to increase from 496 million tons (milled rice) in 2020 to 555 million tons in 2035 and is mainly driven by increasing population and economic growth in developing countries (FAO, 2019). Around 76% of the world’s rice is grown by puddled transplanting (Fageria, 2007; Zhao et al., 2018). However, climate change and limited resources such as water, labor, and energy are putting immense pressure on rice cultivation, especially in puddled transplanted production systems (Chauhan and Mahajan, 2013). Though puddled transplanting provides an almost perfect environment for growing rice through enhanced nutrient availability, ease of seedling establishment, and weed control due to flooded conditions (Sanchez, 1973), it also requires high inputs (Kumar and Ladha, 2011). With the increasing frequency and intensity of droughts due to climate change, puddled transplanted rice is becoming an increasingly unsustainable method of rice cultivation (Chauhan and Mahajan, 2013).

In contrast to puddled transplanting, direct seeding of rice into dry soil (dry-DSR) by drilling or broadcasting, with or without tillage, requires fewer inputs and provides ecosystem benefits. Direct seeding improves soil structure, reduces methane emissions, and supports non-rice crop rotations such as rice-wheat (Kumar et al., 2016). Compared to puddled transplanting, direct-seeded rice cultivation saves 11% to 40% of water (Cabangon et al., 2002; Tabbal et al., 2002), 11% to 66% of labor (Rashid et al., 2009; Kumar and Ladha, 2011), and reduces greenhouse gas emission by as much as 40% to 47% (Pathak et al., 2009; Susilawati et al., 2019). Globally, direct seeding is practiced on about 25% of the total rice cultivated area (Kumar and Ladha, 2011). Driven by the benefits, direct-seeded rice cultivation is gaining momentum in Asia (Singh and Shahi, 2015) and South America, in both upland and rainfed lowland rice ecosystems (Farooq et al., 2011; Sandhu et al., 2015). It is also commonly practiced in Europe and the United States because it saves labor and time through ease of mechanization (Farooq et al., 2011).

While there are many benefits to practicing direct seeding over puddled transplanting, achieving an optimum plant stand is a significant challenge posed by deep (5–8 cm) and/or variable sowing depth under dry-DSR (Ohno et al., 2018), which results in low yields relative to potential and restricts the wider adaptation of dry-DSR (Mahender et al., 2015; Lee et al., 2017). Though deep-sowing under dry-DSR is desirable because it facilitates seeds’ access to moisture for germination when irrigation is unavailable, deep root establishment, and resistance to lodging in the later stages of crop development (Ekanayake et al., 1986), the downside risks of low yields due to poor plant stand are a significant barrier to more widespread adoption by farmers. In contrast to wheat and maize, most rice cultivars are not adapted to deep sowing (Rebetzke et al., 2007; Liu et al., 2017). Thus, breeding rice for adaptation to deep-sowing, including speedy and uniform seedling establishment, is crucial to de-risking dry-DSR, thereby addressing a critical need to produce more rice with fewer inputs, especially less water.

Morphological adaptations to deep sowing in rice include elongated mesocotyls and/or coleoptiles. The mesocotyl in grasses is the first internode of the stem, located between the scutellar and coleoptiler nodes (Lee et al., 2012), and plays a crucial role in seedling emergence. Positive correlations between mesocotyl length, rapid seedling emergence, and early seedling vigor have been documented previously (Mgonja et al., 1988; Luo et al., 2007; Wu et al., 2015a; Sakhale, 2022). The coleoptile is the first leaf, which forms a protective covering around the plumule during germination (Lee et al., 2012), and it contributes to better seedling establishment under flooded or hypoxic conditions under dry-DSR (Ogiwara and Terashima, 2001; Kiyoyuki et al., 2002; Nishimura et al., 2020). Mesocotyl and coleoptile development is regulated by endogenous phytohormones such as cytokinin, gibberellic acid, strigolactones, brassinosteroids, and ethephons (Watanabe et al., 2007; Chen et al., 2014; Hu et al., 2014; Liu et al., 2016).

Prior genome-wide association studies (GWAS) on germplasm panels and genetic mapping studies on biparental populations have been conducted to identify quantitative trait loci (QTLs) for phenotypes associated with deep-sowing tolerance in rice, including mesocotyl length (Wu et al., 2015a; Zhao et al., 2018; Liu et al., 2020), seedling vigor (Guo et al., 2019), and anaerobic germination (Yang et al., 2019; Rohilla et al., 2020; Su et al., 2020). From five GWAS studies of mesocotyl length using rice diversity panels ranging in size from 147 to 621 individuals, ~64 marker-trait associations (18 QTLs and 46 loci) have been reported, with overlap among some trait-associated genomic regions from different studies yielding a minimum of eleven uniquely identified QTL/loci overall (Wu et al., 2015a; Lu et al., 2016; Zhao et al., 2018; Liu et al., 2020; Wang et al., 2021b). Most of the QTLs for mesocotyl length detected in multiple studies were concentrated on chromosomes 1, 3, 6, 9, and 12. However, prior genetic studies of mesocotyl length in rice primarily focused on a particular genetic group (especially *indica*) and often represented germplasm from a particular rice-growing region.

A study of global rice diversity, inclusive of all genetic groups and representing all growing regions, would be desirable to obtain a more comprehensive understanding of traits that confer adaptation to dry-DSR production systems and determine where in the rice germplasm pool and genome desirable alleles for those traits may be most frequently found. Moreover, it would be advantageous to study the genetics of emergence from deep sowing in the field *per se*, in addition to its component traits (e.g., mesocotyl length), as the former is most relevant to farmers. Thus, to further enhance our understanding of the genetics of deep-sowing tolerance in rice, including the identification of genes/QTLs, we performed GWAS analyses with 2.9 million single nucleotide polymorphisms (SNPs) on a combined rice diversity panel of 470 accessions, composed of rice diversity panel 1 (RDP1) (Eizenga et al., 2014) and the aus subset of the 3,000 rice genome project (Li et al., 2014a), representing all of the genetic groups of *O. sativa*. To identify genomic regions associated with dry-DSR adaptive traits that closely represent what occurs in a farmer’s field, we evaluated this broad germplasm panel in the field under deep (8 cm) and shallow control (2 cm) sowing and complemented this with an evaluation of component traits in a controlled environment test-tube experiment (moist germination paper rolled in a test tube for 5 D in the dark at 30°C). We previously reported on the phenotypic diversity of this population and observed that there was large variability among and within the genetic groups for deep-sowing tolerance, noting that aus and aromatic genetic groups were likely especially valuable sources of alleles for deep-sowing tolerance (Sakhale, 2022). In the present study, we sought to identify these advantageous alleles to facilitate more efficient rice improvement *via* marker-assisted selection for dry-DSR adaptation.

## 2 Materials and methods

### 2.1 Plant materials

We studied 470 rice accessions (**Table S1**), with 379 accessions from RDP1 (Eizenga et al., 2014) and a subset of 91 aus accessions from the 3K RGP project (3K RGP, 2014). RDP1 seeds were obtained from the USDA-ARS, Dale Bumpers National Rice Research Center, Stuttgart, Arkansas, Genetic Stocks *Oryza* Collection (GSOR; www.ars.usda.gov/GSOR), and 3K RGP seeds were obtained from the International Rice Germplasm Collection (IRGC) at the International Rice Research Institute (IRRI) in the Philippines. RDP1 is composed of purified accessions of *O. sativa*, including landraces and cultivars collected from 79 countries and 10 geographical regions of the world where rice is grown. The composition of RDP1 includes 33% from East and South-East Asia, 18.3% from South Asia, and 21.5% from the Americas, with the remaining 24% from West and Central Asia, Africa, Europe, and Oceania, and 3.3% of unknown origin. Genetic characterization and sub-population structure of RDP1 have been achieved using simple sequence repeats (SSRs) (Ali et al., 2011) and SNPs (Zhao et al., 2011). RDP1 includes all the major genetic groups of *O. sativa*, including *indica* (21%, n = 79), temperate *japonica* (25%, n = 93), and tropical *japonica* (25%, n = 94), aus (14%, n = 54), *aromatic* cultivars (3%, n = 12), and other admixed (12%, n = 47) (Eizenga et al., 2014). An additional 91 aus accessions from 3K RGP (Li et al., 2014b) were included in the current study to enrich the diversity panel for accessions that are likely to be naturally adapted to dry-DSR production, knowing that aus cultivars are typically grown under upland conditions in India, Bangladesh, and Sri Lanka.

### 2.2 Phenotyping

Two experiments were conducted, one in the field and another in test tubes incubated in a controlled environment. A detailed description of the designs of the two experiments and analyses of phenotypic variation was described in Sakhale (2021) and **Supplementary File S1** and are briefly summarized here. The field experiment with two replications of shallow sowing treatment (shallow control 2 cm) and three replications of deep sowing was conducted in upland farm fields at the International Rice Research Institute (IRRI), Los Baños, Laguna, Philippines (140 10′11.81″N, 1210 15′ 22″ E) with a split-plot design that had main plots of sowing depth treatments (shallow: 2 cm and deep: 8 cm) and sub-plots of accessions (**Table 1**). In the field experiment, the seedling emergence count was recorded daily, starting three days after sowing and continuing until no new emerging seedlings were observed. Based on the emerged seedling count, derived traits such as percent seedling emergence, speed of emergence, and emergence index were estimated (**Table 2**). At 28 days after sowing, up to three emerged seedlings from each plot, if available, were excavated and cleaned under running tap water without disturbing the roots, followed by measurements of mesocotyl length, coleoptile length, shoot length and dry weight, and root length and dry weight (**Table 2**).

**Table 1:** Two experiments, conducted in a field trial at Los Baños, Philippines or in test tubes in a controlled environment chamber, to evaluate a panel of 470 *Oryza sativa* accessions, composed of rice diversity panel 1 (RDP1, n = 379) (Eizenga et al., 2014) and the aus subset (n = 91) of the 3,000 rice genome project (Li et al., 2014a) for adaptation to direct seeding.

S/No.	Experiment	Location	Year	Number of Genotypes	No. of replications	
					Shallow control	Deep stress
1	Variation among rice genotypes for a response to 2 cm (shallow control) or 8 cm (deep stress) sowing in the *Field*	International Rice Research Institute, Los Baños, Philippines	2019	470	2	3
2	Variation among rice genotypes for seedling traits by test-tube method (moist germination paper rolled in a test-tube for 5 D in the dark at a 30°C in a controlled environment chamber)	University of Illinois, Urbana-Champaign	2017	470	4	NA

**Table 2:** Traits analyzed in a genome-wide association study (GWAS) for a panel of 470 *Oryza sativa* accessions, composed of rice diversity panel 1 (RDP1, n = 379) (Eizenga et al., 2014) and the aus subset (n = 91) of the 3,000 rice genome project (Li et al., 2014a).

Trait	Abbreviations	Experiment(s) with trait	Descriptions and notes
Emergence (%)	Emerg	Field	$\text{Emergence(\%)}=\left(\frac{\text{Total seedlings emerged}}{\text{Total seeds sown}}\right)\times 100$
Speed of emergence	SOE	Field	$\text{SOE}=\left(\frac{S_{1}}{t_{1}}+\frac{S_{2}}{t_{2}}+\cdots +\frac{S_{n}}{t_{n}}\right)÷T$ where S_1_, S_2_, and S_n_ are the total number of emerged seedlings time t_1_, t_2_, and t_n_ corresponding to the days after sowing when emergence count was taken. T represents the total number of counting days (Simon et al., 2012).
Shoot dry weight (g)	SDW	Field	Roots and shoots separated by cutting at scutellar node using four-week-old seedlings. The dry weight was recorded on oven-dried (80°C for 72 h) shoot samples.
Mesocotyl length (cm)	ML	Field, test tube	Length of the axis between the scutellar node and coleoptile node, also described as the first internode. ML was measured on four-week-old seedlings.

Traits were measured in a field trial at Los Baños, Philippines in which seeds were planted at 2 or 8 cm deep (control and stress, respectively), or in a test tube experiment after five days of incubation in the dark at 30°C in a controlled environment chamber.

The test-tube experiment was conducted using five seeds per genotype per replication arranged horizontally on moist rolled germination paper that was enclosed in a glass test tube and incubated in the dark at 30°C in a controlled environment chamber for five days (ragdoll method; Shakiba et al., 2017). For the test-tube experiment, four replications in a randomized complete block design were evaluated. After five days of incubation, data were recorded on mesocotyl length, coleoptile length, shoot length, and dry weight, and root length and dry weight (**Table 2**).

### 2.3 Phenotype data analyses

Analyses of variance (ANOVA) were conducted using the lme4 package (Bates et al., 2007) in R software version 3.6.1 (Core Development Team, 2020) for field and test-tube experiments. To estimate the least square means (LS means) for the response variables, a mixed model (Eq. 1) was used to analyze the split-plot design.


(Eq. 1) $${\text{Y}}_{\text{ij}}=\text{μ}+{\text{α}}_{\text{i}}+{\text{β}}_{\text{j}}+\in_{\text{ij}}$$

where Y is the dependent variable at *i*th genotype, *j*th block, μ is the overall mean, α_i_ is a fixed effect due to the *i*th genotype, β_j_ is a random effect due to the *j*th block, ϵ_ij_ is an error term, and α_i_, β_j_, and ϵ_ij_ are independent. LS means were estimated from Eq. 1 using the R package ls means (Lenth and Lenth, 2018).

### 2.4 Genotyping

To enable combined GWAS analyses of RDP1 and the aus subset of 3K RGP, we used 2.9 M SNPs for a subset of 470 accessions selected from the original 5.2 M SNPs for 4,481 accessions of the Rice Reference Panel (RICE-RP). RICE-RP is an imputed dataset between RDP1 and 3K RGP (Wang et al., 2018), and the genotype data for RICE-RP, RDP1, and 3K RGP is publicly available at www.ricediversity.org and https://snp-seek.irri.org. We downloaded the whole RICE-RP genotype dataset during 2019 from https://snp-seek.irri.org and used it as a source for further genotype data sub-setting and analysis. RDP1 genotype data was generated using a high-density rice array (HDRA) comprised of 700,000 SNPs (McCouch et al., 2016). It is one of the highest-density genotyping arrays available for any organism, with a marker density of ~1 SNP per 0.54 Kb. In comparison, the 3,000 rice genome project used whole-genome sequencing with a depth of 14× and the Nipponbare reference genome to obtain ~18.9 million SNPs (Li et al., 2014b). To obtain the genotype dataset (2.9 million SNPs) used in the present study, we filtered the genotype data based on missing sites (<25%) and a minimum minor allele frequency (MAF) of 0.05.

### 2.5 GWAS analyses

To identify marker-trait associations, GWAS analyses were conducted using a mixed linear model (MLM) (Yu et al., 2006) with the R package GAPIT (Lipka et al., 2012). Genotype-based principal component (PC) analysis and kinship (K) matrix were implemented in GAPIT to account for population structure and relatedness among the individuals, respectively, thereby reducing false positives and improving the statistical power of associations. GWAS analyses were performed on three sets of accessions: 1) the whole diversity panel (n = 470), 2) the aus genetic group subset (n = 145), and 3) the aus + *indica* (*INDICA* varietal group) subset (n = 224) (McCouch et al., 2016). The combined diversity panel included the genetic groups aus, *indica*, tropical *japonica*, temperate *japonica*, aromatic, and admixed. The first three principal component analysis scores and K matrix (calculated using the Van Raden method) were fitted as fixed and random effects, respectively (Lipka et al., 2012). The LS means of each accession for each trait were used as a response variable in the fitted GWAS models. False discovery rate adjusted p-values (FDR) (Benjamini and Hochberg, 1995) were estimated in GAPIT, with cutoffs of 0.05 and 0.1 used to identify significant marker-trait associations. A significant peak was defined as a QTL when two or more significant SNPs were found within a 100 kb window ( ± 100 kb) of the lead SNP, whereas a “single SNP genomic region” refers to one significant SNP with no other significant SNPs within 100 kb (Lu et al., 2016; McCouch et al., 2016).

The likely functional annotation of each significant SNP variant was performed using SNPEff 4.3 (Cingolani et al., 2012). A reference genome database in SNPEff for *O. sativa* (IRGSP v5.0) was used for annotation. The predicted effects of significant SNPs were categorized by impact as high (stop gained, frameshift variants), moderate (non-synonymous substitution, missense variants, inframe deletion), low (synonymous substitution), and modifier (exon variant, downstream gene variant) (Cingolani et al., 2012). The candidate genes were selected from a total list of genes obtained *via* annotations of significant SNPs conducted using SNPEff based on the relevance of the biological function of the genes to deep-sowing adaptation traits under DSR. Among the most promising candidate genes were those with biological functions known to have high relevance for adaptation to deep sowing based on prior studies.

## 3 Results

### 3.1 GWAS results for the whole panel, aus, and *INDICA* subset

**Figure 1:**
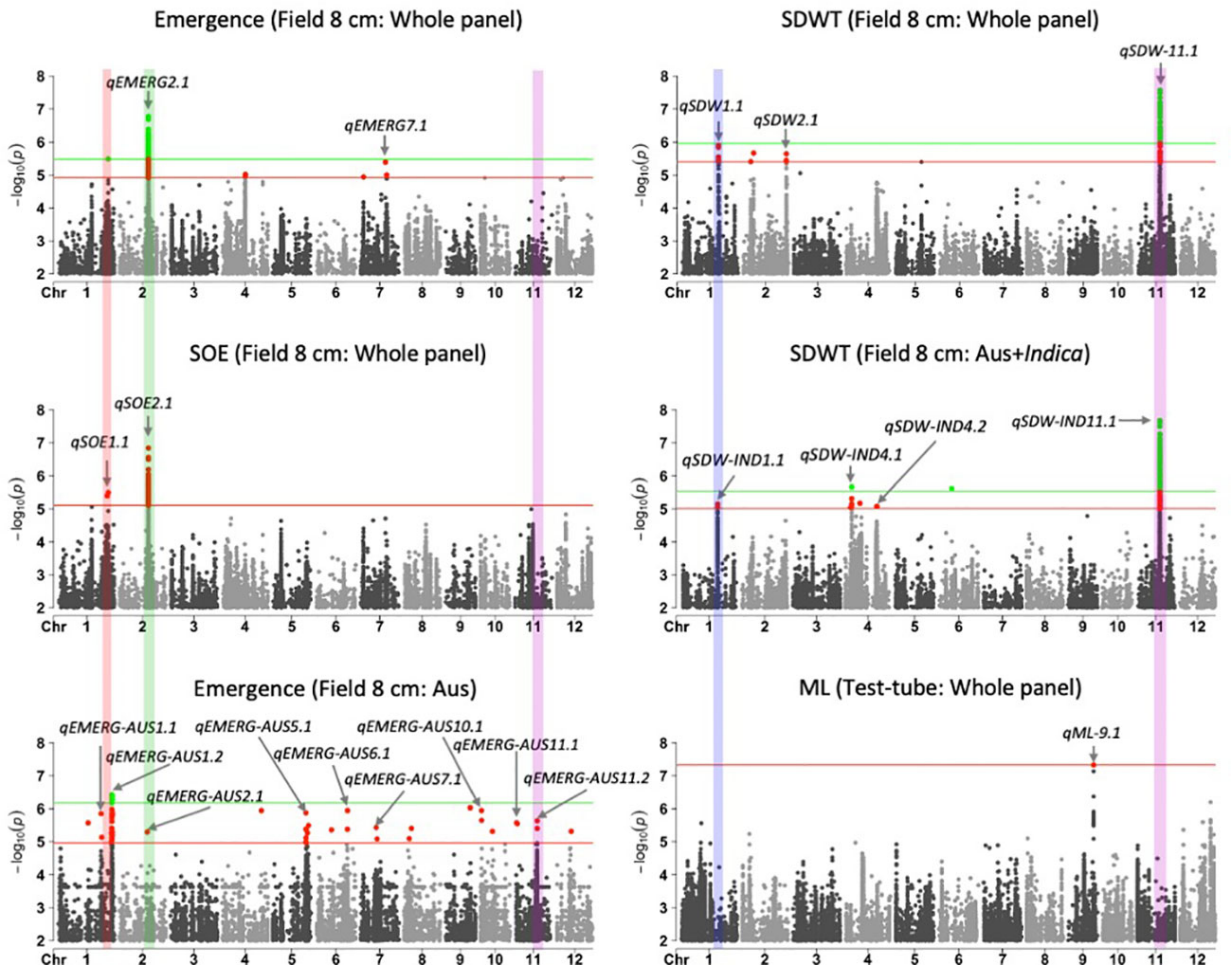
Manhattan plots of results for genome-wide association study (GWAS) analyses based on mixed linear models implemented in GAPIT (Lipka et al., 2012), with principal component (PC) analysis and kinship (K) used to account for population structure and relatedness among individuals, respectively. A panel of 470 *Oryza sativa* accessions, composed of rice diversity panel 1 (RDP1, n = 379) (Eizenga et al., 2014) and the aus subset (n = 91) of the 3,000 rice genome project (Li et al., 2014a), representing all the genetic groups of *O. sativa*, was studied. Traits included emergence (%), speed of emergence (SOE), and shoot dry weight (SDW, g) measured in a field trial at Los Baños, Philippines, in which seed were planted 8 cm deep, and mesocotyl length (ML, cm) measured in a test-tube experiment after five days of incubation in the dark at 30°C in a controlled environment chamber. ‘Whole panel’ indicates SNPs identified from analysis of the whole panel, whereas ‘aus’ was identified from the aus genetic group subset (n = 145) and ‘aus + *indica*’ was identified from the *INDICA* varietal group subset (n = 224). Green dots and green horizontal lines (topmost) represent single nucleotide polymorphisms (SNPs) that are significant at P ≤0.05 after multiple testing correction, whereas red dots and horizontal lines (lower) represent SNPs significant at P ≤0.1 after multiple testing correction. The locations and names of 21 quantitative trait loci are shown with arrows and text. Four vertical bars show overlapping QTLs identified in multiple analyses.

**Figure 2:**
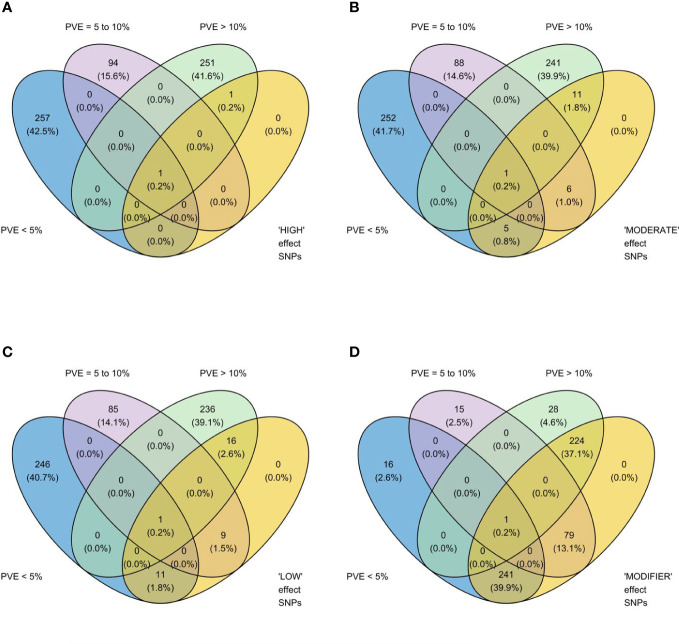
Venn diagrams showing relationships between two methods of estimating the phenotypic impact of 603 significant single nucleotide polymorphisms (SNPs) identified in genome-wide association study (GWAS) analyses of rice (*Oryza sativa*) for four traits that contributed to tolerance to deep-sowing: SNP effect estimated by the software SNPEff (Cingolani et al., 2012) in four categories, **(A)** high, **(B)** moderate, **(C)** low, and **(D)** modifier, and three classes of percent variation explained (PVE), >10% (high), 5 to 10% (moderate), and<5% (low), estimated by genome-wide association study (GWAS) analyses based on mixed linear models implemented in GAPIT (Lipka et al., 2012), with principal component (PC) analysis and kinship **(K)** used to account for population structure and relatedness among individuals, respectively. High impact genes include stop_gained and frameshift_variants; moderate includes missense variants; low includes synonymous variants; and modifiers include exon_variant and downstream_gene_variant (Cingolani et al., 2012). A panel of 470 *O. sativa* accessions, composed of rice diversity panel 1 (RDP1, n = 379) (Eizenga et al., 2014) and the aus subset (n = 91) of the 3,000 rice genome project (Li et al., 2014a), representing all the genetic groups of *O. sativa*, was studied. Traits included emergence (%), speed of emergence, and shoot dry weight (g) measured in a field trial at Los Baños, Philippines, in which seed were planted 8 cm deep, and mesocotyl length (cm) measured in a test-tube experiment after five days of incubation in the dark at 30°C in a controlled environment chamber.

**Figure 3:**
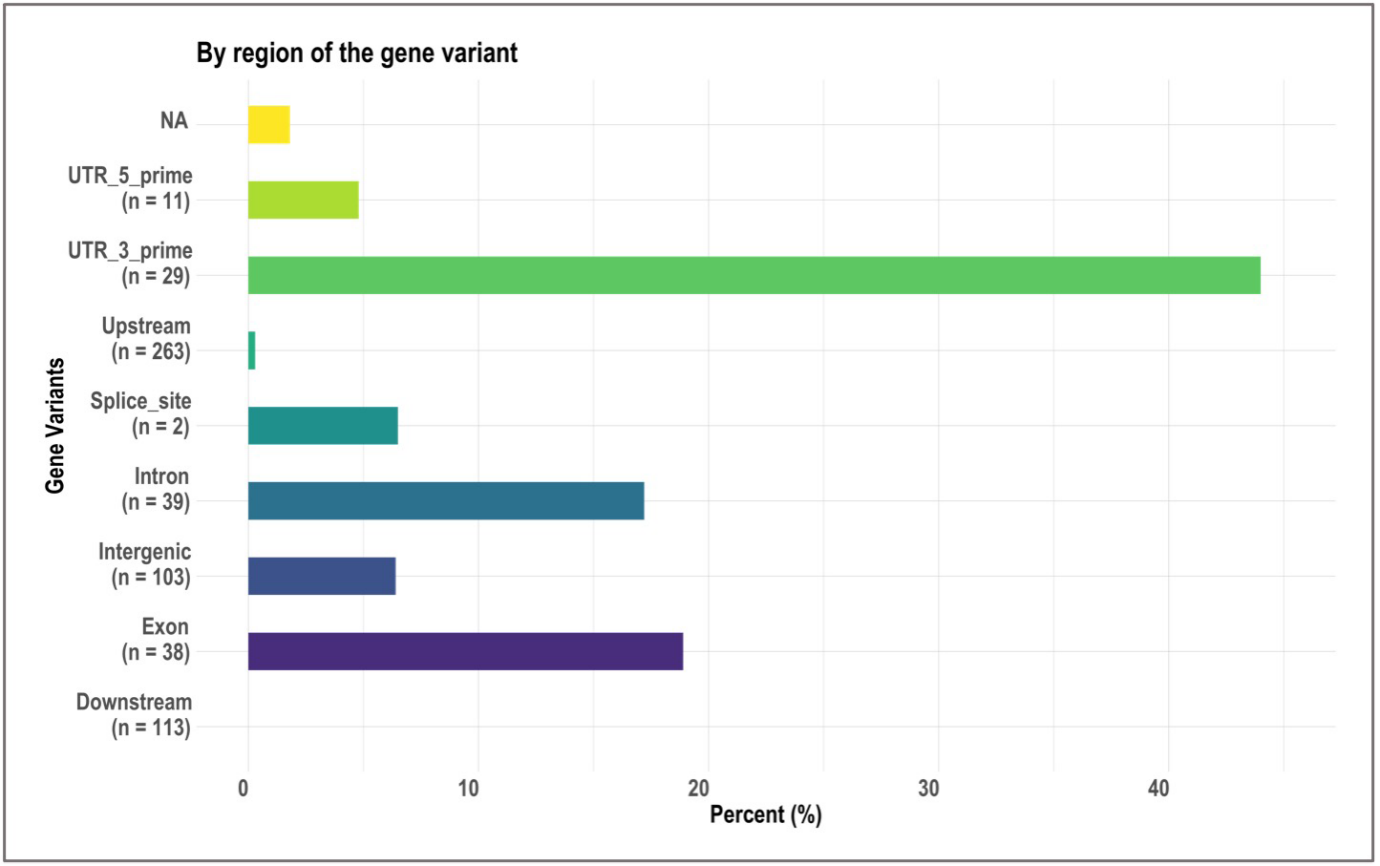
Bar plots showing, by gene region, the relative proportion of 603 significant single nucleotide polymorphisms (SNPs) identified in genome-wide association study (GWAS) analyses of rice (*Oryza sativa*) for four traits that contributed to tolerance to deep-sowing. A panel of 470 *O. sativa* accessions, composed of rice diversity panel 1 (RDP1, n = 379) (Eizenga et al., 2014) and the aus subset (n = 91) of the 3,000 rice genome project (Li et al., 2014a), representing all the genetic groups of *O. sativa*, was studied. Traits included emergence (%), speed of emergence (SOE), and shoot dry weight (SDW, g) measured in a field trial at Los Baños, Philippines, in which seed were planted 8 cm deep, and mesocotyl length (ML, cm) measured in a test-tube experiment after five days of incubation in the dark at 30°C in a controlled environment chamber.

We identified 236 significant SNPs, which included five QTLs at FDR-corrected P ≤0.05 for two traits (emergence (%) and shoot dry weight (g)) under field deep-sowing (**Figure 1**, **Tables 3**, **4**, **Table S1**). While, at FDR-corrected P ≤0.1, we identified 603 SNPs across four traits, including emergence (%, n = 388), speed of emergence (n = 5), shoot dry weight (g, 208) from field deep-sowing, and mesocotyl length (cm, n = 2) from the test-tube experiment, which contributed to tolerance of rice to deep-sowing (**Table S2**). The 603 SNPs included 21 different QTLs (**Table 3**) and 14 single-SNP regions (**Table 4**). Thus, most of the significant SNPs were found in QTL regions. Of the 21 QTL identified, 11 were for emergence (field 8 cm), two for speed of emergence (field 8 cm), seven for shoot dry weight (field 8 cm), and one for mesocotyl length (test tube) (**Figure 1**). Four sets of overlapping QTLs were identified in multiple analyses (set 1: *qSOE-1.1* and *qEMERG-AUS-1.2*; set 2: *qEMERG-2.1*, *qSOE-2.1*, and *qEMERG-AUS-2.1*; set 3: *qEMERG-AUS-11.2*, *qSDW-11.1*, and *qSDW-IND-11.1;* set 4: *qSDW-1.1* and *qSDW-IND-1.1*) (**Figure 1**). Of the 14 single-SNP regions, 12 were unique for emergence (field 8 cm), one was found for both emergence and speed of emergence (field 8 cm), and one was for shoot dry weight (field 8 cm) (**Table 4**). The phenotypic variance explained (PVE) by each of the 603 significant SNPs ranged from 2.6% to 17.8%; 47% of SNPs explained >10% PVE, 10% of SNPs explained 5% to 10%, and 43% of SNPs explained<5% (**Table S2**). Thus, large-effect and small-effect SNPs predominated, whereas moderate-effect SNPs were infrequent. SNP effects estimated by the software SNPEff (Cingolani et al., 2012) included one gene identified as high, 4% as moderate, 6% as low, and 92% as modifiers. Modifier SNPs that also had an estimated PVE of<5% were the most frequent (44%), followed by those with PVEs of >10% (40%) and 5%–10% (8%; **Figure 2**). Notably, one SNP was identified as having high impact and a PVE >10%, and only 10 moderate SNPs had a PVE of 5%–10% (**Figure 2**). Analysis by genomic region found that a large proportion of the SNPs were found upstream (44%), downstream (19%), in intergenic regions (17%), and in introns (7%) of genes (**Figure 3**). About 2% of the significant SNPs were in 5’ untranslated regions (UTRs) and 5% in 3’ untranscribed but potential regulatory regions (**Figure 3**). Exons accounted for 6% of the SNPs, with 21 being synonymous, one nonsense mutation, and 16 missense mutations (**Figure S1**).

**Table 3:** Quantitative trait loci (QTL; n = 21) for emergence (EMERG, %), speed of emergence (SOE) and shoot dry weight (SDW, g) were measured in a field trial at Los Baños, Philippines, in which seeds were planted 8 cm deep, and for mesocotyl length (ML, cm) measured in a test-tube experiment after five days incubation in the dark at 30°C in a controlled environment chamber.

QTL	Lead SNP	Chr.	Pos. (Mb)	FDR adjusted P-value	PVE	Candidate gene	Reported Locus
*qSDW-1.1*	mlid0006464909	1	28.2–28.2	0.05	2.9–3.1	Os01g0684900	
*qSDW-IND-1.1*	mlid0006467086	1	28.2–28.2	0.08	8.7–8.9	Os01g0684900	
*qEMERG-AUS-1.1*	mlid0007349026	1	32.8–33.3	0.05	13.6–15.9	OsCKX5, Os01g0775500	
*qSOE-1.1*	mlid0008395331	1	37.6–38.4	0.05	2.7–2.8	FSM	qML1.4 (Xiong et al., 2017; Wang et al., 2021b)
*qEMERG-AUS-1.2*	mlid0008927838	1	40.9–41.7	0.03	13.2–17.8	OsHAK6, Os01g0938600, Os01g0938900, Os01g0939200, Os01g0939300, GPDH2, Os01g0939700, CKX4, OsHXK3, OsPR2, OsMUS81, OsNPC2	qMel-1 (Cao et al., 2002; Wu et al., 2015a), and seq-rs609 (for shoot length) (Dang et al., 2014; Lu et al., 2016)
*qEMERG-AUS-2.1*	mlid0014164785	2	20.9–22.6	0.05	3.0–14.0	Os02g0556100, OsCPT1, Os02g0586500	
*qEMERG-2.1*	mlid0014379250	2	22.4–22.6	0.04	2.6–3.9	Os02g0584800, Os02g0585100	
*qSOE-2.1*	mlid0014379250	2	22.5–22.6	0.04	2.6–3.7	Os02g0584800, Os02g0585100	
*qSDW-2.1*	mlid0016678899	2	33.8–33.9	0.07	2.8–3.0	Os02g0796000	
*qSDW-IND-4.1*	mlid0026230401	4	5.3–5.3	0.04	8.8–10.0	Os04g0177300, Os04g0177400	
*qSDW-IND-4.2*	mlid0030902086	4	24.5–24.7	0.09	9.0–9.0	Os04g0492300	
*qEMERG-AUS-5.1*	mlid0038926320	5	25.8–27.9	0.05	13.1–16.0	HSP101, Os05g0519900, Os05g0520200, OsIAA18, OsGT43E	seq-rs2638 (Lu et al., 2016)
*qEMERG-AUS-6.1*	mlid0045621374	6	24.5–24.6	0.05	14.4–16.2	OsCYP93F1, OsFTIP1	
*qEMERG-AUS-7.1*	mlid0049755165	7	11.9–12.6	0.05	13.4–14.6	Os07g0300900-OsRH5	qFML7.1 (Zhao et al., 2018; Liu et al., 2020)
*qEMERG-7.1*	mlid0051610551	7	19.0–20.0	0.05	2.6–2.9	Os07g0519100, Os07g0519300	
*qML-9.1*	mlid0065562882	9	19.7–19.8	0.05	3.0–3.0	Os09g0510000, Os09g0510200, OsCCR3, HMGR3	
*qEMERG-AUS-10.1*	mlid0066458929	10	1.1–1.2	0.05	15.3–16.2	OsXTH27, Os10g0117400, ZRP4, Os10g0118800	
*qEMERG-AUS-11.1*	mlid0072207246	11	1.4–1.9	0.05	15.0–15.0	Os11g0141100	
*qEMERG-AUS-11.2*	mlid0076143945	11	17.4–17.4	0.05	14.5–15.2	OsWRKY72	
*qSDW-11.1*	mlid0076168099	11	17.0–17.6	0.01	3.0–15.0	OsSPL19, OsWRKY72, OsENODL22, Os11g0491600, Os11g0492300	
*qSDW-IND-11.1*	mlid0076168461	11	17.3–17.6	0.02	8.6–14.3	OsSPL19, OsWRKY72, OsENODL22, Os11g0491600, Os11g0492300	

QTL, quantitative trait loci; Chr, chromosome; pos, position (Mb); PVE, phenotypic variance explained (%).

Significant single nucleotide polymorphisms (SNPs) identified by genome-wide association study (GWAS) analyses based on mixed linear modelsimplemented in GAPIT (Lipka et al., 2012), with principal component (PC) analysis and kinship (K) used to account for population structure and relatedness among individuals, respectively. A genomic region was defined as a QTL if two or more significant marker-trait association (SNPs) were found in a 100 kb window around the lead SNP. A panel of 470 Oryza sativa accessions, composed of rice diversity panel 1 (RDP1, n = 379) (Eizenga et al., 2014) and the aus subset (n = 91) of the 3,000 rice genome project (Li et al., 2014a), representing all the genetic groups of O. sativa, was studied. QTL without a suffix were identified from analysis of the whole panel, whereas those with the AUS suffix were identified from the aus genetic group subset (n = 145) and ‘IND’ from the aus + indica (INDICA varietal group) subset (n = 224). The candidate genes in each QTL region were identified using annotations on lead SNP of the QTL, considering the genes that falls under 100 kb window of lead SNP.

**Table 4:** Trait-associated single nucleotide polymorphisms (SNPs; n = 14) identified by genome-wide association study (GWAS) analyses based on mixed linear models implemented in GAPIT (Lipka et al., 2012), with principal component (PC) analysis and kinship (K) used to account for population structure and relatedness among individuals, respectively.

Significant SNP	Chr.	Pos. (Mb)	FDR adjusted P-value	PVE	Trait	Env	Candidate gene	Reported locus
mlid0000953939	1	4.7	0.07	14%	Emergence	Field (8 cm): aus	Cytokinin dehydrogenase 1 (OsCKX1)	
mlid0005023858	1	21.7	0.08	13%	Emergence	Field (8 cm): aus	OsMST7: sugar transport protein	
mlid0005272164	1	22.7	0.05	15%	Emergence	Field (8 cm): aus	OsWRKY77: positive regulator of disease resistance in plants	
mlid0005934063	1	25.6	0.08	3%	Emergence, SOE	Field (8 cm): whole panel	UGT703A1 (Glycosyltransferase (GT): plays essential role in cell wall biosynthesis	
mlid0011051684	2	8.5	0.07	3%	SDW	Field (8 cm): whole panel	tRNA-Gln: glutamyl-tRNA (Gln) amidotransferase subunit A, chloroplastic/mitochondrial	*qML-2* (Huang et al., 2010; Liu et al., 2020)
mlid0029436447	4	17.8	0.09	3%	Emergence	Field (8 cm): whole panel	OsWAK43: wall associated kinase gene.	
mlid0031961058	4	30.4	0.05	16%	Emergence	Field (8 cm): aus	VLN4: Villin-4 modulation of polar auxin transport	
mlid0047351824	7	2.0	0.09	3%	Emergence	Field (8 cm): whole panel	OsSWAP70B: protein serine/threonine kinase activity	
mlid0054688205	8	4.1	0.08	13%	Emergence	Field (8 cm): aus	Os08g0170700: disease resistance protein (RP)	*qLOE-8* (Zhang et al., 2006; Liu et al., 2020)
mlid0055075459	8	5.7	0.05	15%	Emergence	Field (8 cm): aus	Os08g0200100: fatty acid biosynthesis process	
mlid0068889536	10	9.5	0.05	14%	Emergence	Field (8 cm): aus	Os10g0334500: dirigent protein	
mlid0080165050	12	4.7	0.09	3%	Emergence	Field (8 cm): whole panel	Os12g0192500: thiamin-phosphate pyrophosphorylase	
mlid0081609259	12	9.9	0.08	13%	Emergence	Field (8 cm): aus	NRTP1: necrotic root tip 1 (NRTP1)	
mlid0081913997	12	11.1	0.05	14%	Emergence	Field (8 cm): aus	Os12g0288600: conserved hypothetical protein	

QTL, quantitative trait loci; Chr, chromosome; pos, position (Mb); PVE, phenotypic variance explained (%).

The annotated gene/genes within the 100 kb window of significant SNP with biological function relevant to deep-sowing adaptation traits are considered as candidate genes. A panel of 470 Oryza sativa accessions, composed of rice diversity panel 1 (RDP1, n = 379) (Eizenga et al., 2014) and the aus subset (n = 91) of the 3,000 rice genome project (Li et al., 2014a), representing all the genetic groups of O. sativa, was studied. Traits included emergence (%), speed of emergence (SOE) and shoot dry weight (SDW, g) measured in a field trial at Los Baños, Philippines in which seed were planted 8 cm deep. ‘Whole panel’ indicates SNPs identified from analysis of the whole panel, whereas ‘aus’ were identified from the aus genetic group subset (n = 145).

### 3.2 Genomic regions unique or in-common among the whole panel, aus, and *INDICA*

Of the 603 significant SNPs, 257 were identified from analysis of the whole panel, 187 from analysis of the aus subset (emergence), 159 from *INDICA* (shoot dry weight), and 28 were common between the whole panel and *INDICA* for shoot dry weight (**Figure 1** and **Table S2**). Eight of the 21 QTLs were identified in the whole panel, with two for emergence, two for speed of emergence, three for shoot dry weight, and one for mesocotyl length (**Figure 1**, **Table 3**). However, the greatest number of QTLs were found in the aus genetic group, with nine for emergence under field deep sowing (8 cm) (**Figure 1**, **Table 3**). Four QTLs related to shoot dry weight (field 8 cm) were detected in the *INDICA* varietal group (**Figure 1**, **Table 3**).

Of the 21 QTLs identified, eight were from the whole panel, nine were from the aus genetic group, and four were from the *INDICA* varietal group (**Figure 1**). Of the nine QTLs identified in aus, six were unique to this genetic group (*qEMERG-AUS-1.1*, *qEMERG-AUS-1.2*, *qEMERG-AUS-5.1*, *qEMERG-AUS-6.1*, *qEMERG-AUS-7.1*, and *qEMERG-AUS-10.1*) (**Figure 1**, **Table 3**). For the whole panel, three of the eight QTLs were uniquely found in the full set analyses (*qSOE-1.1*, *qSDW-2.1*, and *qEMERG-7.1*). Similarly, of the four QTLs identified in the *INDICA* varietal group, two unique for shoot dry weight were identified under field deep-sowing (*qSDW-IND-4.1* and *qSDW-IND-4.2*) (**Figure 1**, **Table 3**). Notably, greater PVE was observed for the lead SNPs of the QTLs that were unique to the aus genetic group (for emergence) and *INDICA* varietal group (for shoot dry weight) as compared to the QTLs for these traits that were detected in the whole panel (**Table 3**). Two QTLs were identified in both aus and the whole panel (*qEMERG-AUS-2.1*, *qSOE-2.1*, and *qEMERG-2.1* and *qEMERG-AUS-1.2* and *qSOE-1.1*), one QTL was found in aus, *INDICA* and the whole panel (*qEMERG-AUS-11.2*, *qSDW-IND-11.1*, and *qSDW-11.1*), and one QTL was detected in both *INDICA* and the whole panel (*qSDW-IND-1.1* and *qSDW-1.1*). Of the 14 single-SNP regions, nine were unique to aus and five were unique to the whole panel.

### 3.3 Candidate genes and functional gene categories

A total of 156 candidate genes were identified using the SNPEff program (Cingolani et al., 2012) by performing functional annotation on the 603 significant SNPs identified in the GWAS analysis (**Tables S1**, **S2**). About half of the candidate genes were identified from aus exclusively, and all but one of these were for emergence under deep sowing in the field (**Table S3**). Among the traits, emergence accounted for 74% of the candidate genes. Analysis of the whole panel identified 35% of the candidate genes, and 26% were exclusively from *INDICA*. Of the 156 candidate genes, information on the underlying biological function was available from prior studies for 53 (**Table S3**). The subset of 53 candidate genes with biological information was grouped into 15 categories based on the type of biological function associated with the candidate gene and its relevance to deep-sowing tolerance (**Tables 5**, **6**). Among the top five gene categories, phytohormone biosynthesis/signaling ranked first with 11 genes, followed by cell wall biosynthesis (n = 7), abiotic stress response (n = 6), biotic stress response (n = 6), and detoxification (n = 4) (**Table 5**). Twelve of the 53 candidate genes included synonymous SNP variants (alleles), and seven included missense SNP variants (**Table S3**).

**Table 5:** Categorization of 53 candidate genes with known biological function that included or were within a 100 kb window of a significant single nucleotide polymorphisms (SNPs) detected in a genome-wide association study (GWAS) for a panel of 470 *Oryza sativa* accessions, composed of rice diversity panel 1 (RDP1, n = 379) (Eizenga et al., 2014) and the aus subset (n = 91) of the 3,000 rice genome project (Li et al., 2014a).

#	Gene category	Candidate genes	Total number of genes
1	Phytohormone biosynthesis/signaling	*Os01g0938900, Os01g0939200, OsNPC2, OsCPT1, OsIAA18, FSM, OsCKX1, CKX4, OsCKX5, VLN4*, *OsWRKY72*	11
2	Cell wall biosynthesis and defense response	*UGT703A1, Os01g0938600, OsWAK43, OsGT43E, OsCCR34, OsXTH27, Os09g0510000*	7
3	Abiotic stress response	*Os02g0796000, Os04g0177400, OsSWAP70B, HSP101, Os10g0334500, OsFTIP1*	6
4	Biotic stress response	*GPDH2, OsPR2, Os11g0129000, OsWRKY77, ZRP4, Os08g0170700*	6
5	Detoxification	*Os02g0584800, Os02g0585100, Os01g0684900, Os01g0939700*	4
6	Nutrient uptake	*Os07g0519100, Os07g0519300, OsHAK6*	3
7	Protein synthesis	*tRNA-Gln, Os05g0519900, Os05g0520200*	3
8	Sugar transport/metabolism	*OsMST7, OsHXK3, Os12g0192500*	3
9	Cellular development	*OsSPL19, Os04g0177300*	2
10	DNA damage response/DNA repair	*Os01g0939300, OsMUS81*	2
11	Metabolite modification	*Os11g0141100, OsCYP93F1*	2
12	mRNA splicing	*Os02g0586500*	1
13	Transcription	*Os04g0492300*	1
14	Lipid biosynthesis	*Os08g0200100*	1
15	rRNA processing	*OsRH5*	1
		Total	53

Traits included emergence (%), speed of emergence measured and shoot dry weight measured in a field trial at Los Baños, Philippines in which seed were planted 8 cm deep, and mesocotyl length (ML, cm) measured in a test tube experiment after five days incubation in the dark at 30°C in a controlled environment chamber.

**Table 6:** Frequency of candidate genes, by gene category and trait, that included or were within a 100 kb window of a significant single nucleotide polymorphisms (SNPs) detected in a genome-wide association study (GWAS) for a panel of 470 *Oryza sativa* accessions, composed of rice diversity panel 1 (RDP1, n = 379) (Eizenga et al., 2014) and the aus subset (n = 91) of the 3,000 rice genome project (Li et al., 2014a).

Gene category	Emerg	SOE	SDW	ML	Total
Phytohormone biosynthesis/signaling	10 + 1*		1*		11
Cell wall biosynthesis and defense response	5 + 1*	1*		1	7
Abiotic stress response	4		2		6
Biotic stress response	6				6
Detoxification	2 + 1*	1*	1		4
Nutrient uptake	3				3
Protein synthesis	2		1		3
Sugar transport/metabolism	3				3
Cellular development			2		2
DNA damage response/DNA repair	2				2
Metabolite modification	2				2
Other	4				4
Total	43 + 3*	2*	6 + 1*	1	53

*Same gene identified for two or more traits.

QTL, quantitative trait loci; Chr, chromosome; pos, position (Mb); PVE, phenotypic variance explained (%).

Traits included emergence (%), speed of emergence measured and shoot dry weight measured in a field trial at Los Baños, Philippines in which seed were planted 8 cm deep, and mesocotyl length (ML, cm) measured in a test tube experiment after five days incubation in the dark at 30°C in a controlled environment chamber.

## 4 Discussion

### 4.1 Aus was an outstanding genetic resource for identifying QTLs and genes that conferred adaptation to dry-DSR

In aus, we identified the greatest number of unique QTLs and candidate genes associated with traits that conferred adaptation to deep-sowing, which was consistent with our prior results that aus was the best genetic group in rice for the emergence and mesocotyl length under deep-sowing (Sakhale, 2022). To the best of our knowledge, this is the first GWAS on the aus genetic group of rice for deep-sowing tolerance. The *INDICA* varietal group, which included *indica* and aus genetic groups, had four significant QTLs uniquely associated with shoot dry weight under field deep sowing. Aus cultivars are traditionally grown in Bangladesh and India under upland rainfed conditions. Aus is typically direct-seeded or broadcast prior to the start of the monsoon rainy season (Maclean et al., 2013; Shelley et al., 2016). Thus, aus evolved under selection pressure for tolerance to abiotic stresses, with the tradeoff that most cultivars are low yielding. In contrast, the high-yielding green revolution cultivars of rice adapted to the tropics belong to the *indica* genetic group. Thus, a key lesson of the current study is that aus is an excellent genetic resource for improving high-yielding *indica* rice with alleles for greater adaptation to deep-sowing and dry-DSR production systems. The QTLs and candidate genes identified in the current study can be directly used by rice breeders to develop improved cultivars that are high yielding but use less water than current modern cultivars.

### 4.2 Promising candidate genes identified

We identified a total of twelve especially promising candidate genes. Of the 12, 11 were under the phytohormone category, which was further grouped into five categories: cytokinin (CK), auxin, gibberellic acid (GA), jasmonic acid (JA), and meristem morphology. The last candidate gene among the twelve was a high-effect (stop gained and missense variant) candidate gene of unknown function that was detected under deep sowing. Ten of 12 candidate genes under the phytohormone genes category were identified in aus for four traits (emergence, shoot dry weight, and mesocotyl length), including three genes that regulated cytokinin (*CKX4*, *OsCKX5*, and *OsCKX1*), four auxins (*VLN4*, *OsWRKY72*, *OsCPT1*, and *OsIAA18*), two gibberellic acids (*OsNPC2* and *Os01g0939200*), and one jasmonic acid (*Os01g0938900*, **Tables 3**, **4**). The meristem morphology gene, *flattened shoot meristem* (*FSM*), was identified for emergence in an analysis of the whole panel in the test tube experiment (**Table 3**). The gene *OsWRKY72*, which is involved in the auxin transport pathway, was also detected in *INDICA* varietal group for shoot dry weight under deep sowing. The high effect gene (*Os11g0491600*) with an unknown function was detected for shoot dry weight in the whole panel and redetected in INDICA varietal group under deep sowing.

Given that phytohormone biosynthesis/signaling were the largest categories of candidate genes identified in this study, our results support the conclusions of prior studies that phytohormones and their relative proportions play an essential role in the process of germination and seedling emergence, mainly through regulation of cell division and cell elongation (Feng et al., 2017; Xiong et al., 2017). Such regulation is especially critical when seeds are sown deeply under dry-DSR and the shoot must elongate sufficiently to reach the surface. Prior studies have documented that mesocotyl length is moderately to strongly correlated with emergence under deep sowing, thereby playing an important role in seedling emergence, especially for deeply sown seeds (Mgonja et al., 1988; Luo et al., 2007; Chung, 2010; Wu et al., 2015a; Lu et al., 2016; Lee et al., 2017; Ohno et al., 2018; Sakhale, 2022). Moreover, mesocotyl elongation is inhibited in response to light (Feng et al., 2017). Exposure to light (red light > far-red light > blue light) results in decreased auxin in epidermal cells and ultimately halts mesocotyl elongation (Vanderhoef and Briggs, 1978). Also, upon light exposure, mesocotyl elongation is inhibited through dynamic endogenous phytohormone changes, mainly due to decreased IAA, trans-zeatin (tZ), and GA_3_, and increased jasmonic acid (Feng et al., 2017). Our study has identified specific phytohormone genes and alleles in rice that appear to confer adaptation to deep sowing, and these may have direct application to rice improvement.

**Figure 4:**
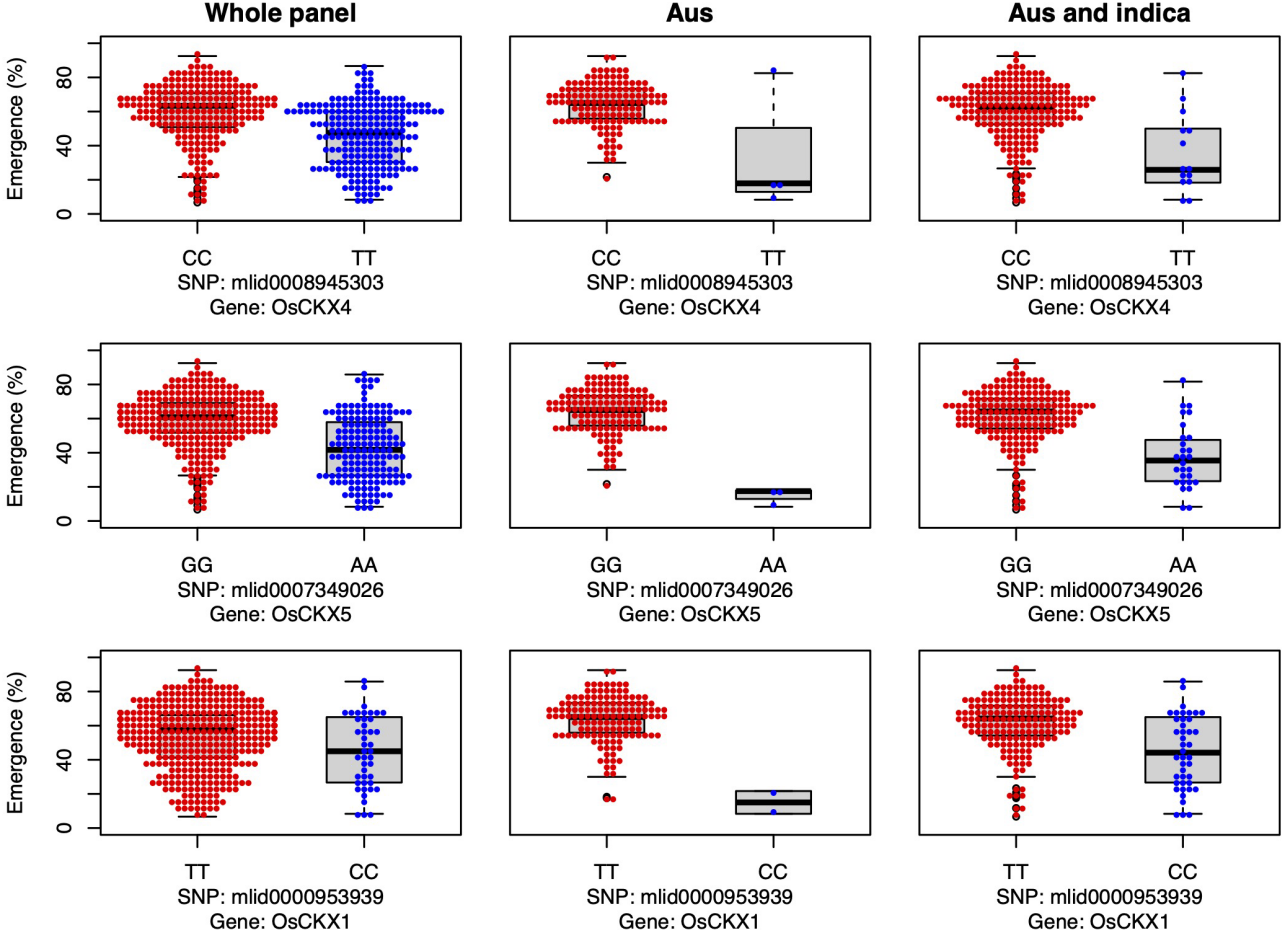
Allelic effects of three cytokinin dehydrogenase candidate genes for seedling emergence (%; y-axis) of deeply sown (8 cm) dry direct-seeded rice (*Oryza sativa*), phenotyped in a field trial at Los Baños, Philippines. Major alleles are shown on the left and minor alleles on the right. A panel of 470 accessions, composed of rice diversity panel 1 (RDP1, n = 379) (Eizenga et al., 2014) and the aus subset (n = 91) of the 3,000 rice genome project (Li et al., 2014a), representing all the genetic groups of *O. sativa*, was studied. Shown are results for the whole panel, the aus genetic group subset (n = 145), and the aus + *indica (INDICA* varietal group) subset (n = 224). In boxplots, the bold line indicates the median, the lower and upper edges of the box represent the 25th and 75th percentiles, and whiskers extend to the most extreme data, excluding outliers. Each point overlaying the boxplots represents an individual accession’s mean value.

Three cytokinin dehydrogenase/oxidase (CKX) genes were associated with seedling emergence under field deep sowing in the aus subset of the current study (*CKX4*, *OsCKX5*, *OsCKX1*; **Figure 4**). Cytokinins (CK) regulate plant growth and development, including the promotion of stem elongation by increasing the rate and number of cell divisions in the shoot meristem (Werner et al., 2001). Elongation of the rice mesocotyl in the dark is promoted by cytokinin (Hu et al., 2014). CKX is the main enzyme that irreversibly inactivates cytokinin in plants, thereby regulating this key phytohormone. CKX is a multi-gene family in plants, and eleven paralogs have been annotated in rice (Schmülling et al., 2003; Yeh et al., 2015). Disruption of CKX genes results in increased cytokinin content and, thus, greater stem elongation (Duan et al., 2019; Zhang et al., 2021). Our study revealed that the most frequent aus and *indica* alleles of three CKX genes promoted seedling emergence under deep sowing *via* longer mesocotyls and/or longer overall shoots (**Figure 4**). The fact that we found three of the 11 rice CKX loci to be associated with emergence from deep sowing is evidence of the importance of this gene family for adaptation to dry DSR. Further validation and optimization of these and potentially other CKX genes in rice *via* gene editing or a transgenic approach could enable their application to improve rice adaptation to deep sowing.

**Figure 5:**
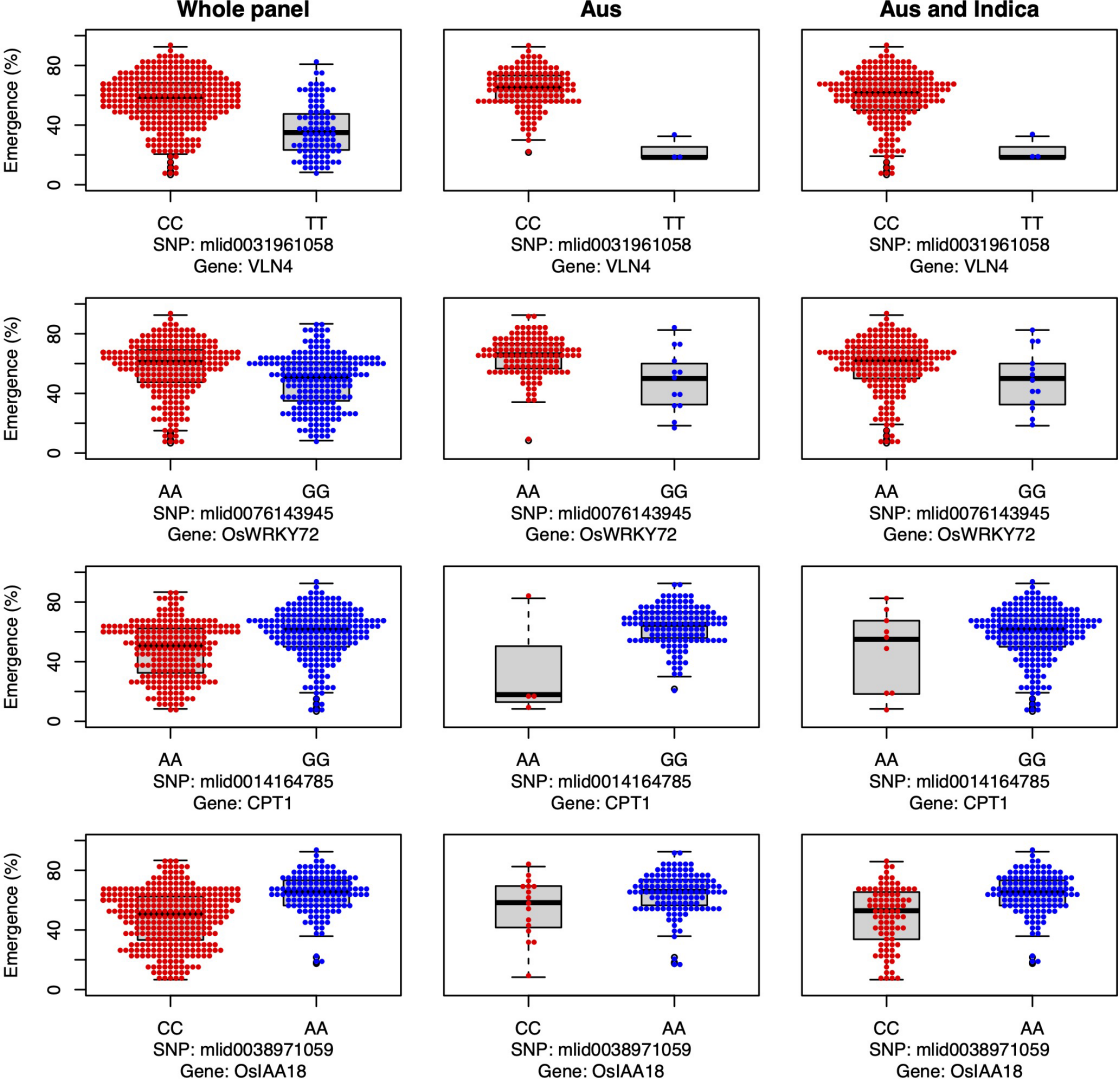
Allelic effects of four auxin transport and signaling candidate genes for seedling emergence (%; y-axis) of deeply sown (8 cm) dry direct-seeded rice (*Oryza sativa*), phenotyped in a field trial at Los Baños, Philippines. Major alleles are shown on the left and minor alleles on the right. A panel of 470 accessions, composed of rice diversity panel 1 (RDP1, n = 379) (Eizenga et al., 2014) and the aus subset (n = 91) of the 3,000 rice genome project (Li et al., 2014a), representing all the genetic groups of *O. sativa*, was studied. Shown are results for the whole panel, the aus genetic group subset (n = 145), and the aus + *indica (INDICA* varietal group) subset (n = 224). In boxplots, the bold line indicates the median, the lower and upper edges of the box represent the 25th and 75th percentiles, and whiskers extend to the most extreme data, excluding outliers. Each point overlaying the boxplots represents an individual accession’s mean value.

Additionally, we identified four candidate genes involved in auxin transport that were associated with emergence in aus and shoot dry weight in *INDICA* under field deep sowing (*VLN4*, *OsWRKY72*, *OsCPT1*, and *OsIAA18*; **Figure 5**). Auxins are indole ring-based, simple small molecules that play an essential role in plant growth and development, including cell differentiation, cell division, and cell elongation (Xu and Xue, 2012). Auxin activity relies on polar transport (Benjamins and Scheres, 2008). The VLN4 gene that we identified as a candidate is involved in bundling actin filaments and changing the dynamics of actin in the cortical array, thereby directly affecting auxin polar transport and, consequently, plant morphogenesis (Wu et al., 2015b; Zou et al., 2019). Similarly, candidate genes *OsWRKY72* and *OsIAA18* are transcription factors involved in auxin transport and signaling, respectively (Sahebi et al., 2018; Wang et al., 2021a). *OsIAA18* has been shown to contribute to drought tolerance in rice *via* the auxin transport and abscisic acid (ABA) signaling pathways (Wang et al., 2021a). Lastly, the candidate gene *OsCPT1* is responsible for the lateral translocation of auxin from the light-exposed side of the rice coleoptile to the darker side, thereby promoting cell elongation on the dark side and causing the shoot to bend towards the light (Haga et al., 2005). A similar mechanism could also be happening under deep sowing of rice, as the tip of the shoot is closer to the light source, and translocation and accumulation of auxin in the lower part of the stem could promote cell elongation leading to emergence from deep sowing. In *OsCPT1* and *OsIAA18*, we observed that the minor allele of each gene favored increased seedling emergence (**Figure 5**), and thus they could be potential targets for marker-assisted selection to develop rice cultivars with improved tolerance to deep sowing.

**Figure 6:**
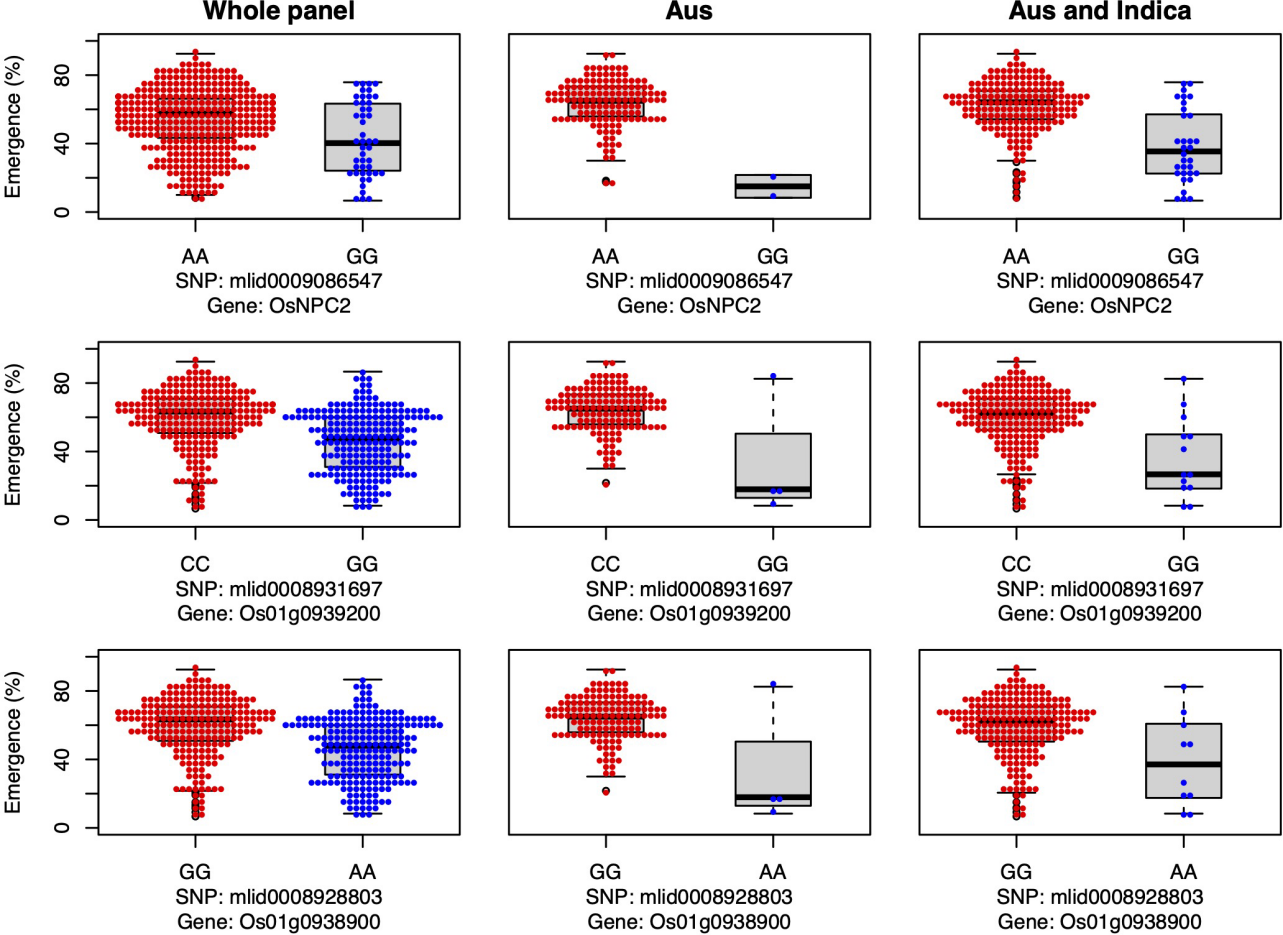
Allelic effects of two gibberellic acid and one jasmonic acid candidate gene for seedling emergence (%; y-axis) of deeply sown (8 cm) dry direct-seeded rice (*Oryza sativa*), phenotyped in a field trial at Los Baños, Philippines. Major alleles are shown on the left and minor alleles on the right. A panel of 470 accessions, composed of rice diversity panel 1 (RDP1, n = 379) (Eizenga et al., 2014) and the aus subset (n = 91) of the 3,000 rice genome project (Li et al., 2014a), representing all the genetic groups of *O. sativa*, was studied. Shown are results for the whole panel, the aus genetic group subset (n = 145), and the aus + *indica (INDICA* varietal group) subset (n = 224). In boxplots, the bold line indicates the median, the lower and upper edges of the box represent the 25th and 75th percentiles, and whiskers extend to the most extreme data, excluding outliers. Each point overlaying the boxplots represents an individual accession’s mean value.

The genes *OsNPC2* and *Os01g0939200* have been previously shown to be involved in GA signaling, and in the current study both genes were associated with emergence under field deep sowing in aus (Yang et al., 2021) (**Figure 6**). For each gene, the most common allele in aus and *INDICA* was associated with greater seedling emergence under deep sowing (**Figure 6**). Yang et al. (2021) previously documented that *OsNPC2* contributes to GA-mediated mesocotyl length in rice. Wang et al. (2009) found that *Os01g0939200* is responsible for ethylene and GA signaling. Indeed, exogenous GA application has been found to strongly stimulate the elongation of the mesocotyl and coleoptile in rice (Watanabe et al., 2007; Watanabe et al., 2018), which is consistent with our observations that these two GA-related genes confer increased seedling emergence under deep-sowing.

We identified a JA-related candidate gene, *Os01g0938900*, associated with seedling emergence under field deep-sowing in aus (**Figure 6**). The gene *Os01g0938900* is a transcription factor and plays a role in JA biosynthesis (Guo et al., 2018). JA is necessary for plant growth and development and antagonizes the biosynthesis of GA, resulting in the inhibition of stem elongation (Met al., 2013). Ethylene-mediated repressed of JA biosynthesis promotes greater mesocotyl and coleoptile elongation in rice seedlings (Xiong et al., 2017). JA also interacts with other hormone signaling pathways such as auxins, ABA, salicylic acid (SA), and GAs to regulate plant growth and stress response (Yang et al., 2019a). Thus, crosstalk between JA and other phytohormones maintains the required hormonal balance, contributing to adaptive mesocotyl length and better emergence under deep sowing in dry-DSR.

**Figure 7:**
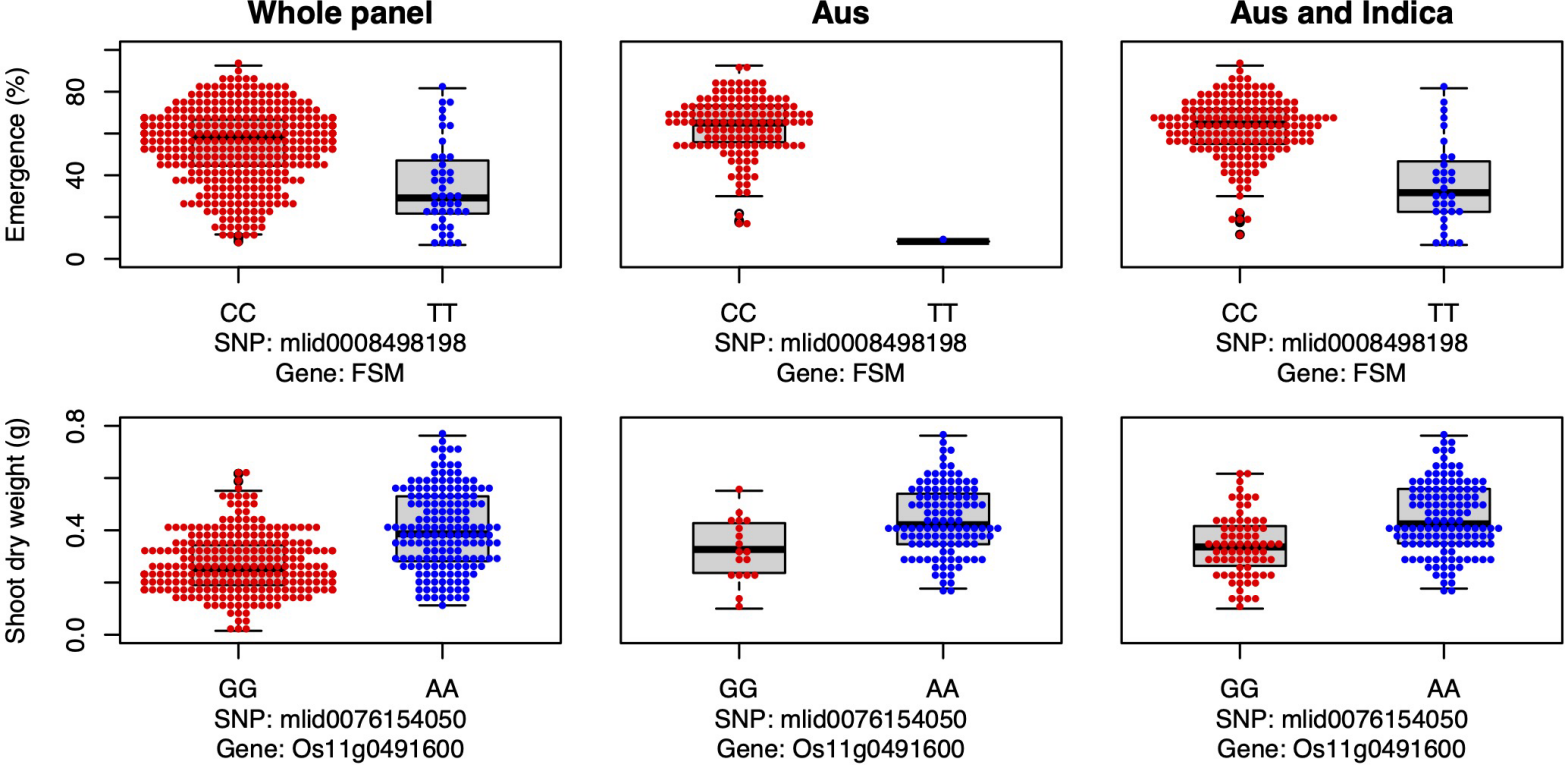
Allelic effects of a flattened shoot meristem (FSM) and a high-effect (Os11g0491600) candidate genes for seedling emergence (%; y-axis) and shoot dry weight (g; y-axis) respectively, of deeply sown (8 cm) dry direct-seeded rice (*Oryza sativa*) phenotyped in a field trial at Los Baños, Philippines. Major allele is shown on the left and the minor allele on the right. A panel of 470 accessions, composed of rice diversity panel 1 (RDP1, n = 379) (Eizenga et al., 2014) and the aus subset (n = 91) of the 3,000 rice genome project (Li et al., 2014a), representing all the genetic groups of *O. sativa*, was studied. Shown are results for the whole panel, the aus genetic group subset (n = 145), and the aus + *indica (INDICA* varietal group) subset (n = 224). In boxplots, the bold line indicates median, the lower and upper edges of the box represent the 25th and 75th percentiles, and whiskers extend to the most extreme data, excluding outliers. Each point overlaying the boxplots represents an individual accession’s mean value.

Candidate gene *FSM* was associated with seedling emergence in the whole panel under deep sowing (**Figure 7**), and it encodes the p150 subunit of chromatin assembly factor 1, which has a critical role in the shoot apical meristem maintenance *via* regulation of the duration of S and G2 phases (Abe et al., 2008). Thus, *FSM* is critical for shoot growth, including the growth of mesocotyls. In our study, the major allele was advantageous for emergence from deep sowing under dry-DSR (**Figure 7**).

Lastly, a high-effect candidate gene (*Os11g0491600*) of an unknown conserved hypothetical protein was associated with shoot dry weight detected under deep sowing in the whole panel and *INDICA* varietal group. *Os11g0491600* had high-impact variants, including stop-gained, premature stop-codon, missense, and synonymous variants (alleles), explaining 11% to 12% PVE (**Table 3**, **Table S1**). The most frequent/mutant allele was associated with greater shoot dry weight under deep sowing in aus and *INDICA* (**Figure 7**). Greater shoot dry weight is an indicator of high seedling vigor, and it has been reported in prior studies that it plays a critical role not only in emergence from deep sowing but also can improve the post-emergence competition with weeds under direct seeding of rice (Haque et al., 2007; Wang et al., 2010; Dimaano et al., 2017; Zhang et al., 2017; Anandan et al., 2020). High seedling vigor is also known to be associated with mesocotyl elongation under deep sowing (Mgonja et al., 1988) and is critical for early seedling establishment under DSR (Anandan et al., 2020).

## 5 Conclusion

Most of the beneficial alleles for seedling emergence under deep sowing that we identified were from aus, which is typically a low-yielding but stress-tolerant genetic group of rice. Thus, it would be desirable to introgress these advantageous aus alleles for adaptation to deep-sowing under dry-DSR into high-yielding modern cultivars of *indica* (for the tropics) and *japonica* (for temperate latitudes). Development of one or more multiparent advanced generation intercross (MAGIC) breeding populations to combine high seedling emergence under deep-sowing *via* shoot elongation from aus, tolerance to low oxygen during germination from tropical *japonica* (which can be highly advantageous when deeply sown rice seeds receive unexpected early rains), and high-yield potential from *indica*, would likely be a successful strategy for breeding improved cultivars that are well-adapted to dry-DSR and for making long-term genetic gains. Moreover, additional work to optimize the most compelling candidate genes associated with seedling emergence, especially those involved in phytohormone biosynthesis/signaling, could be a promising strategy to make rapid genetic gains for dry-DSR adaptation. The sub-population-specific QTLs identified in this study will be useful for molecular breeding and gene cloning. Though the current study has identified many potentially useful QTL and genes for adaptation to deep sowing, there is great potential to mine additional genes by screening larger panels of individual rice genetic groups, especially aus, *indica* and tropical *japonica*. Lastly, given that the dynamic balance between the endogenous phytohormones (CK, auxins, GA, and JA) at the seedling stage plays a crucial role in deep-sowing tolerance under dry-DSR, there is the potential to develop a seed priming method that uses synthetic plant growth regulators for improving mesocotyl length and seedling emergence under deep sowing.

## Data availability statement

The original contributions presented in the study are included in the article/**Supplementary Material**. Further inquiries can be directed to the corresponding author.

## Author contributions

SS conceptualized and designed the study, conducted the statistical analyses, and wrote the manuscript with input and critical revisions from ES and AK. SY helped in conducting the field experiment and provided feedback on the manuscript. AL and LC helped in the GWAS analysis and provided expertise in interpreting the results. All authors contributed to the article and approved the submitted version.
