# Supplementary material for: Genome-wide association analysis for emergence of deeply sown rice (Oryza sativa) reveals novel aus-specific phytohormone candidate genes for adaptation to dry-direct seeding in the field
Source: Front Plant Sci. 2023 Jun 12;14:1172816. doi: 10.3389/fpls.2023.1172816 (PMC10291202; doi:10.3389/fpls.2023.1172816)
Supplement: Supplementary file 1 [file DataSheet_1.pdf]

Supplementary File S1: Details of plant material, phenotype experiments, cultivation practices, and traits recorded.

A detailed description of the designs of the two experiments, treatments within experiments, traits recorded, and analyses of phenotypic variation was described in Sakhale (2021) and are briefly summarized here for the convenience of the readers.

1 Plant materials

We studied 470 *O. sativa* accessions (genotypes), with 379 from Rice Diversity Panel 1 (RDP1; Eizenga et al., 2014) and a subset of 91 aus accessions from the 3000-rice genome project (Li et al., 2014). The germplasm panel included representation from admixed, admixed-*indica*, admixed-*japonica*, aromatic, aus, *indica*, temperate-*japonica*, tropical-*japonica*, and unknown. To conduct the experiments, the seeds for RDP1 were obtained from International Rice Research Institute's International Rice Germplasm Collection (IRGC) in, Los Baños, Philippines, and the USDA/ARS Genetics Stocks-Oryza (GSOR) collection. Seeds of the 3K RGP were obtained from the International Rice Germplasm Collection.

2 Phenotyping

Two experiments (field and test tube) on seedling growth were conducted on the full germplasm panel (Supplementary Table S1). A field experiment was conducted at IRRI, Los Baños, Philippines, with two planting depths: 2 cm (shallow) and 8 cm (deep), whereas the test-tube experiment was conducted in a controlled environment chamber at the University of Illinois Urbana-Champaign, IL, USA.

2.1 Experiment 1 (Field): Variation among rice genotypes for response to 2 cm (shallow control) or 8 cm (deep stress) sowing in the field

Field experiment compared two sowing depth treatments, 2 cm (shallow) and 8 cm (deep), for 470 rice accessions from the germplasm panel. The main plots were planting depth treatments and the subplots were rice accessions. There were two replications of the shallow and three replications of the deep sowing treatments. The field experiment was conducted at upland farm field (non-flooded aerobic soil; 39% clay, 21% sand, and 36% silt) at IRRI in Los Baños, Philippines (140 10′ 11.81″ N, 1210 15′ 22″ E) during the 2019 dry season (planted on 25th January 2019). The experiment field was prepared with standard tillage practices and laser

leveling using a laser-equipped leveling machine to minimize any variation in seeding depth. Tractor-driven till-seeder (T760, Duncan Ag, Australia) was used for planting two sowing depth treatments. Based on availability 30 to 40 dry seeds/plot were used for sowing. Prior to sowing, to break the dormancy seeds were exposed to 50 °C for three days in a hot-air oven. A basal dose of fertilizer 100:40:40 N: P_2O_5:K_2O was applied at the time of sowing using the tillage seeder. Subplots were single 3m long rows with 0.2 m between rows. Based on weather conditions and crop stage, surface irrigation was applied when the tip of the young leaf starts rolling in the morning or upon the development of hairy cracks in the surface soil. The excess water was drained immediately after irrigation by opening the bunds to prevent flooded conditions.

Recorded data on ten traits putatively associated with adaptation to dry-DSR. Counted the emerged seedlings daily starting from 3 DAS. Seedling counting continued until 12 DAS or until no new seedlings emerged. At 28 DAS, we excavated three seedlings per subplot using a shovel ensuring the roots were undamaged, and after cleaning roots, root length, mesocotyl length, and shoot length were measured using a ruler (cm). Following length measurements, the Root and shoot were separated and oven dried at 80 °C for 48 h for measuring dry weight. Five random plants from the remaining 2 m row were harvested at maturity to record the grain yield.

2.2 Experiment 2 (Test-tube): Variation among rice genotypes when germinated in test-tubes containing rolled moist germination paper (ragdolls) for 5 d in the dark at 30 °C in a controlled environment chamber.

In a test-tube experiment, we evaluated the germplasm panel (n=470) for seedling traits root, shoot, mesocotyl, and coleoptile length. The experiment was a randomized complete block design, with four replications in time. The present test-tube experiment protocol was heavily based on the 'ragdoll' seed germination method described by (Shakiba et al., 2017). To make ragdolls we used heavy-weight (34.5 kg) seed-germination papers (15.24 × 25.4 cm) (Model: SD7615L, Anchor Paper Co., Saint Paul, MN, USA) that was first soaked in an aqueous suspension of 100 mg/liter benomyl (Methyl 1-butylcarbamoyl-2-benzimidazolecarbamate, Sigma-Aldrich Chemistry, St. Louis, MO, USA) for 10 minutes. Seeds were surface sterilized in 10 ml of 2.6% sodium hypochlorite for 10 min, followed by three time rinsing with sterilized

deionized water. Five surface-sterilized seeds per replication per accession were horizontally arranged in the center on moistened germination paper leaving equal distance (12.5 cm) on both sides. Keeping the seeds firmly on the germination paper, the paper was rolled carefully to make the ragdoll and placed in a labeled 25 x 150 mm glass test-tube containing 10 ml of distilled water. The test-tube was subsequently closed with closure caps and further wrapped with parafilm (Zoro Parafilm #: G4455333). Sets of 72 tubes were placed in a tray, and each tray was labeled with the time and date before placement inside an incubator (Model 818, Thermo Scientific Precision, Asheville, NC, USA) that maintained a constant 30±1 °C in the dark. After five days of incubation, trays were stored at 4 °C. Germinated seeds were measured for root, shoot, mesocotyl, and coleoptile length.

References

Sakhale, S.A. (2021). Rice adaptation to high temperature at flowering stage and direct-seeding. https://hdl.handle.net/2142/115733 (Doctoral dissertation, University of Illinois Urbana-Champaign)

Shakiba, E., Edwards, J.D., Jodari, F., Duke, S.E., Baldo, A.M., Korniliev, P., McCouch, S.R., & Eizenga, G.C. (2017). Genetic architecture of cold tolerance in rice (Oryza sativa) determined through high resolution genome-wide analysis. *PLoS ONE*, *12*, e0172133. https://doi.org/10.1371/journal.pone.0172133
