# Supplementary figures and images for: Genome-wide association analysis for emergence of deeply sown rice (Oryza sativa) reveals novel aus-specific phytohormone candidate genes for adaptation to dry-direct seeding in the field
Source: Front Plant Sci. 2023 Jun 12;14:1172816. doi: 10.3389/fpls.2023.1172816 (PMC10291202; doi:10.3389/fpls.2023.1172816)

## By type of gene variant

Gene Variants

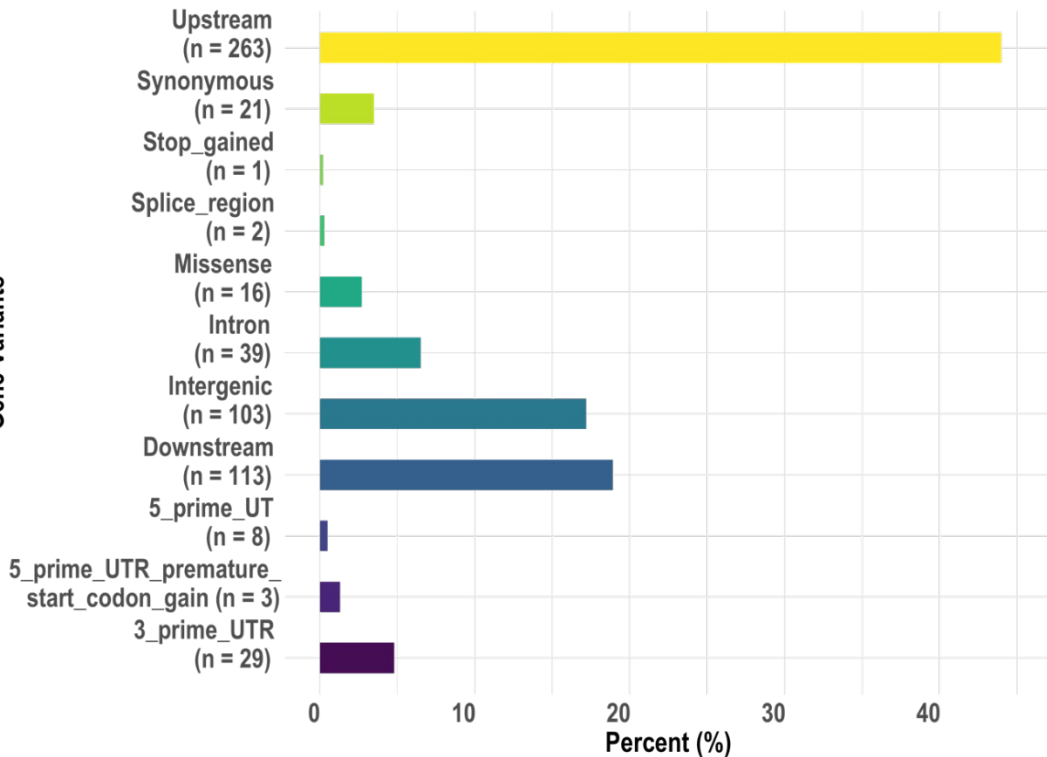

Supplement: Supplementary file 2 [file DataSheet_2.pdf]
